# NURSING HOME EXPECTATIONS AND MENTAL HEALTH AMONG OLDER ADULTS IN THE HEALTH AND RETIREMENT STUDY

**DOI:** 10.1093/geroni/igad104.1930

**Published:** 2023-12-21

**Authors:** Annalise Lane, Linh Dang, Briana Mezuk

**Affiliations:** Department of Veterans Affairs, Ann Arbor, Michigan, United States; University of Michigan, Ann Arbor, Michigan, United States; University of Michigan, Ann Arbor, Michigan, United States

## Abstract

Three million older adults live in long-term care facilities. Transitioning into a nursing home is a major life event that can worsen mental health. Expectedness of nursing home transitions may have implications for mental health; however, this area is understudied. This study examined the relationship between expectations of transitioning into a nursing home in the next five years and depressive symptoms and passive suicidal ideation, and how social integration modified these relationships. Sample included respondents aged 65+ from the 2018 Health and Retirement Study (n=7,944, mean age=73.9, 55.4% female, 80.5% Non-Hispanic White). Nursing home transition expectations were modeled continuously (range: 0-100%). Mental health outcomes were assessed by the Composite International Diagnostic Interview-Short Form. Social integration measured composition, frequency of contact, and closeness with spouses/partners, children, other family members, and friends. Multivariate logistic regressions were fit for depressive symptoms and passive suicidal ideation separately, adjusting for demographic, health characteristics, and health-related reasons for moving. Additional models simultaneously adjusted for all social integration measures. Higher nursing home expectations were associated with elevated depressive symptoms (OR: 1.06, 95% CI: 1.01-1.11), major depressive episode (OR: 1.08, 95% CI: 1.02-1.15), and suicidal ideation (OR: 1.10, 95% CI: 1.03-1.16). These relationships persisted even after accounting for social integration. Future research can explore modifiable factors salient to these relationships, such as perceived social support and family care, to inform mental health-targeted interventions as older adults transition into long-term care.

